# Family Aggregation and Risk Factors in Phobic Disorders over Three-Generations in a Nation-Wide Study

**DOI:** 10.1371/journal.pone.0146591

**Published:** 2016-01-19

**Authors:** Hans-Christoph Steinhausen, Helle Jakobsen, Andrea Meyer, Povl Munk Jørgensen, Roselind Lieb

**Affiliations:** 1 Research Unit for Child and Adolescent Psychiatry, Psychiatric Hospital, Aalborg University Hospital, Mølleparkvej 10, 9000, Aalborg, Denmark; 2 Clinical Psychology and Epidemiology, Department of Psychology, University of Basel, Basel, Switzerland; 3 Department of Child and Adolescent Psychiatry, University of Zurich, Zurich, Switzerland; 4 Department M, Aarhus University Hospital, Risskov, Denmark; Peking University, CHINA

## Abstract

**Objective:**

This nation-wide register-based study investigated how often phobic disorders (PHO) and co-morbid disorders occur in affected families compared to control families. Furthermore, the study addressed the impact of sex, year of birth, and degree of urbanization in terms of risk factors.

**Method:**

A total of N = 746 child and adolescent psychiatric participants born between 1969 and 1986 and registered in the Danish Psychiatric Central Research Register (DPCRR) with a diagnosis of a mental disorder before the age of 18, and developed PHO at some point during their life-time until a maximum age of 40 years were included. In addition, N = 2229 controls without any diagnosis of mental disorders before age 18 and that were matched for age, sex, and residential region were included. Diagnoses of mental disorders were also obtained from the first- degree relatives as a part of the Danish Three Generation Study (3GS). A family load component was obtained by using various mixed regression models.

**Results:**

PHO occurred significantly more often in case than in control families, in particular, in mothers and siblings. Substance use disorders (SUD), Depressive disorders (DEP), anxiety disorders (ANX) and personality disorders (PERS) in the family were significantly associated with specific phobia in the case-probands. After controlling for various mental disorders comorbid to PHO it was found that some of the family transmission could be caused by various other mental disorders in family members rather than the PHO itself. Female sex and more recent year of birth were further risk factors while region of residence was not related to the manifestation of PHO. Case-relatives did not develop PHO earlier than control relatives. After adjusting for various additional explanatory variables, the family load explained only 0.0013% of the variance in the manifestation of PHO in the case-probands

**Discussion:**

These findings, based on a very large and representative dataset, provide evidence for the family aggregation and further risk factors in PHO. In contrast to anxiety disorders and other major mental disorders the family load of PHO in this nation-wide study was rather low.

## Introduction

Phobic disorders (e.g., specific phobia, social phobia, agoraphobia) are among the most commonly occurring mental disorders in children, adolescents, and adults [1–7] and belong to the disorders with the earliest onset [8]. Population based studies revealed substantial comorbidity between phobic disorders and other mental disorders [9–15].

One risk factor for phobic disorders is a positive family history of phobic disorders. Numerous studies suggest that phobic disorders are familial. Specifically in the case of social phobia, results from family studies suggest that relatives of clinical probands with social phobia have an increased risk of social phobia compared to relatives of individuals without social phobia [16–20]. One family study evaluated whether DSM-III-R phobic disorders (simple phobia, social phobia, agoraphobia) aggregate in families [21]. This study confirmed moderate specific familial aggregation of each of the DSM-III-R phobic disorders. Comparably, studies that used the high-risk approach reported consistently an association between parents and offspring for social phobia [22,23] and agoraphobia [24]. Some of these studies also suggest that the offspring of phobic parents have an increased risk not only for phobic disorders, but also for other mental disorders, such as other forms of anxiety disorders, affective disorders or substance use disorders [22]. These findings suggest some degree of co-transmission of phobic disorders and other mental disorders.

Currently, it is not yet conclusive whether demographic risk factors play a role in phobic disorders as in other mental disorders. Studies by Peen et al. [25] and Dekker et al. [26] suggest an increased risk for anxiety disorders in urban versus rural living people. While month of birth has been considered as a risk factor for schizophrenia [27], bipolar disorders [28], and anxiety disorders other than phobic disorders [29], it is unknown whether it can also be conceptualized as risk factor for phobic disorders.

The present register-based study evaluates the familial aggregation of phobic disorders (PHO) as part of the Danish three generation study. We provide another report in a series of matched, case-control, population-based analyses of three-generation family aggregation and associated risk factors of mental disorder [27–32]. One previous report has already dealt with an overall assessment of family aggregation across all anxiety disorders, but excluded ICD-10 phobic disorders [29]. In the present study, we aim to apply these analyses to phobic disorders (PHO). To our knowledge, this is the first effort to demonstrate familial aggregation of PHO using a three generation approach based on a nation-wide psychiatric case registry. Specifically, we explore: (1) the family load of PHO and other mental disorders in families with an affected proband compared to families of non-ill controls, and (2) the effects of further putative risk factors, i.e., sex, year of birth, and degree of urbanization on proband-case status.

## Materials and Methods

### Description of the dataset

This case-control study was based on coded registry data and was approved by the Danish Data Protection Agency, National Board of Health, and Statistics Denmark. The dataset contained 746 case-probands with PHO, identified through the Danish Psychiatric Central Research Registry (DPCRR)[33,34]. The DPCRR contains data on all individuals entering the public mental health system. From 1969 to 1994 only inpatient admissions were registered whereas both in- and outpatient admissions have been recorded since 1995. Case-probands were born between 1969 and 1986. They received any ICD-10 diagnosis [35] before age 18 and had developed PHO before the maximum age of 40 years.

In Denmark, each individual is given an individual number at birth in the Danish Central Civil Registration Register (DCR), thereby making it possible to cross- identify each person in various other country-wide registers. In this way, for each case-proband, three control-probands were identified in the DCR, yielding a total of 2229 using risk-set sampling; that is, each were alive and without registrations in the DPCRR at the time of case-proband ascertainment during childhood and adolescence, and were matched to case-probands on age (same year and month of birth), sex, and region of residence at the index time which is the time of first admission to psychiatry. Control-probands were excluded if they received any psychiatric diagnosis before age 18 but kept if they received a diagnosis of PHO starting at age 18 up to a maximum age of 40 years. Due to matching restrictions, not all case-probands had three control-counterparts for the analyses to be reported. Collectively, the case- and control-participants are referred to as probands.

Family members were identified through the DCR and DPCCR as previously described [31]. Lifetime data were obtained since the first registration of any mental disorder and the maximum period of observation for the diagnostic ascertainment of relatives via the DPCRR was 40.69 years. Registry diagnoses of PHO were defined according to ICD-8 criteria (code 300.2) until 1994, then, as of 1995, according to ICD-10 criteria (code F40). To study the role of other mental disorders in the family aggregation, the following additional diagnoses were also considered in the analyses: substance use disorders (SUD, ICD-8: 303, 304; ICD-10: F1), schizophrenia (SCZ 295; F20), bipolar disorders (BP 296; F30-31), depression (DEP 300.49; F32-33, 34.1), anxiety disorders (ANX, 300.0; F41, F93.0–93.2), eating disorders (ED 306.5; F50), and personality disorders (PERS, 301; F60-61.0).

### Statistical analyses

Fisher's exact tests were used for the comparison of frequencies of mental disorders in the relatives of case-probands compared to relatives of controls. Effect sizes were assessed by Cramer`s *V*, where coefficients ranging from 0 to 0.1 are considered very small, from 0.1 to 0.3 small, from 0.3 to 0.5 medium, and ≥0.5 large. Odds ratios (OR) were calculated to determine whether PHO occurred more often in the relatives of case probands compared to relatives of controls. PHO in family members was only counted if the PHO diagnosis appeared before the PHO diagnosis in the proband.

Conditional logistic regression was applied to determine whether the illness status of family members increased the risk of the disease in the case probands more strongly than in the control-probands. The indicator variables examined were family PHO and other mental disorders in family members. If data from a family member were missing the value of the variable was 0, indicating the family member was assumed to be unaffected. Since this method takes matching into account, the matched variables were not included in the risk analysis. All variables were included as categorical variables.

Multinomial logistic regression analysis was used for the comparison of risk factors originating from the various mental disorders in family members in three different groups: case-probands with PHO and other comorbid mental disorders, case-probands with pure PHO (i.e. without any comorbidity), and the group of control-probands without PHO. The group of control-probands was used as the reference group, i.e. the presence of family disorders in both the comorbid PHO group and the pure PHO group were compared to the controls. The risk was measured as relative risk ratios (RRR).

Mixed logistic regression was used to estimate a family load measured as a random effect showing the dependence among family members in relation to how often each family developed PHO. A model including a random effect is used when a dataset is divided into groups—in this case into families. In the model, each family has its own intercept and the random effect is the estimated standard deviation (SD) in the intercept on the logarithmic scale. For instance, consider a mixed logistic regression model for a matched case-control study with families of three generations which has a random effect of SD = 0.5. This means that members of a family which is one standard deviation above the mean have the odds of getting PHO which are 65% [since exp(0.5) = 1.65] higher than members of an average family.

The random effect was examined by group (namely, cases and controls). Furthermore, the regression analysis included the matched explanatory variables, i.e., sex, year of birth, month of birth, and region of residence at the index time of the case-probands. The latter was converted into a dichotomous variable comparing the capital of Copenhagen to all other regions. Sex, month of birth, and region of residence were included as categorical variables while year at birth was included as a continuous variable.

Cox regression with shared frailty was applied to investigate if case family members developed PHO earlier than control family members, i.e. the probands were excluded from the analysis. A Cox model is a survival model and it is a function of the hazard rate which is the risk over time of experiencing a certain event such as PHO. A Cox model with shared frailty includes a random effect named a frailty which describes the effect of unknown elements not included as parameters in the model. The family load component is estimated as a random effect (frailty), i.e. it describes the dependence or lack of such between the families of the study. The frailty does not vary within families, but rather between these.

A frailty measures the dependence among the family members in relation to the time to disease onset implying that a family with a high value of the frailty developed PHO earlier than a family with a small value of the frailty. The frailty is assumed to follow the gamma distribution with a mean value of one and variance zero. The purpose of this approach is to estimate the effect of the explanatory variables while also estimating zero. The analysis includes the matched explanatory variables sex, year and month of birth, and region of residence. The latter was converted into a dichotomous variable comparing the capital of Copenhagen to all other regions. Sex, month of birth and region of residence were included as categorical variables whereas year of birth was included as a continuous variable. All analyses were carried out by using the statistical software programs Stata version 13.0 [36] and R version 3.0.2 [37].

## Results

Sample sizes, sex distributions, and the observation periods in the case- and control populations are shown in Table 1. Only persons with a PHO diagnosis were included in the calculation of mean age at diagnosis of PHO for both probands and family members in case and control families. In both case and control-populations the maximum observational period was 40.69 years. For the case-probands, the mean observation time amounted to 27.67 years.

**Table 1:** Demographic characteristics of the subjects in case and control families.

		N (%)		Age at diagnosis	Observation time in years*		
	Total	Males	Females	mean (SD)	Mean	SD	Range
Case families							
Probands	746	246 (32.98)	500 (67.02)	20.62 (4.70)	27.67	3.67	19.05–40.61
Fathers	736	736 (100)	0 (0)	-	39.61	4.04	7.31–40.69
Mothers	743	0 (0)	743 (100)	44.52 (5.44)	40.07	3.10	12.95–40.69
Siblings	873	423 (48.45)	450 (51.55)	23.35 (6.46)	28.72	7.50	0.01–40.69
Offspring	108	50 (46.30)	58 (53.70)	-	8.91	3.61	0.25–21.02
Total	3206	1455 (45.38)	1751 (54.62)	20.84 (5.17)	32.94	8.81	0.01–40.69
Control families							
Probands	2229	735 (32.97)	1494 (67.03)	23.99 (3.41)	27.78	3.69	18.63–40.61
Fathers	2191	2191 (100)	0 (0)	50.01 (6.50)	39.92	3.30	10.73–40.69
Mothers	2226	0 (0)	2226 (100)	38.43 (12.66)	40.33	2.25	12.41–40.69
Siblings	2941	1466 (49.85)	1475 (50.15)	21.01 (5.78)	27.60	7.50	0.00–40.69
Offspring	384	191 (49.74)	193 (50.26)	-	8.78	3.14	0.50–25.50
Total	9971	4583 (45.96)	5388 (54.04)	30.70 (13.04)	32.47	9.11	0.00–40.69

The frequencies of PHO in families of case and control-probands by class of relative are shown in Table 2. Overall, relatives (in the sense of any family members) of case-probands had a 2.71-fold increased odds for PHO when compared to relatives of control-probands. More specifically, mothers of case-probands had a 3.01-fold increased odds for PHO before the proband’s PHO diagnosis when compared to mothers of control probands. However, the confidence interval includes the value of one so the possibility of an OR equal to one cannot be ruled out. Siblings of case-probands had a 3.39-fold increased odds when compared to siblings of control-probands. PHO was not significantly different among fathers but the groups were too small to say anything definite and there were no PHO in the offspring of the two samples. All effect sizes were very small.

**Table 2:** Distribution of phobias in case and control families.

	Case N(%)		Control N(%)		Case vs. control (family members only)				
	PHO not present	PHO present	PHO not present	PHO present	OR	Chi2	p	95% CI	V
Probands	0 (0.00)	746 (100)	2225 (99.82)	4 (0.18)					
Fathers	736 (100)	0 (0.00)	2189 (99.91)	2 (0.09)	0.00	0.67	n.s.	-	0.02
Mothers	737 (99.19)	6 (0.81)	2220 (99.73)	6 (0.27)	3.01	4.00	<0.05	0.97–9.38	0.04
Siblings	867 (99.31)	6 (0.69)	2935 (99.80)	6 (0.20)	3.39	5.01	<0.05	1.09–10.53	0.04
Offspring	108 (100)	0 (0.00)	384 (100)	0 (0.00)	-	-	-	-	-
Total	2448 (76.36)	758 (23.64)	9953 (99.82)	18 (0.18)	2.71	6.92	<0.05	1.25–5.86	0.03

As Table 3 shows, the vast majority of case-probands had comorbid disorders, most commonly with PERS followed by DEP and ANX and still quite frequently with SUD, ED, and SCZ but only rarely by BD. Information on the frequencies of the comorbid mental disorders by class of relative across the two proband groups is provided in Table 4. The disorders among family members are only counted if the diagnosis is made before the case-proband of the family receives the first PHO diagnosis. Also for the family members of the control-probands the diagnosis must be made before the date of PHO of the matched case-proband. With the exception of eating disorders, all other mental disorders in total were significantly more common among the case-families compared to the control-families. In contrast to the other family members, fathers were showing comparable rates of schizophrenia, bipolar disorders, and anxiety disorders in the two samples. The offspring had no mental disorders before the PHO diagnosis of the case-proband. For all comparisons the effect sizes were very small to small.

**Table 3:** Distribution of diagnoses among case and control-probands.

	N(%)	
	Case	Control
Probands with pure phobia	127 (17.02)	1 (0.04)
Probands with comorbid phobia	619 (82.98)	3 (0.13)
Other disorders in probands		
Substance use disorders	104 (13.94)	13 (0.58)
Schizophrenia	67 (8.98)	8 (0.36)
Bipolar disorder	22 (2.95)	2 (0.09)
Depression	228 (30.56)	57 (2.56)
Anxiety disorders	215 (28.82)	14 (0.63)
Eating disorders	77 (10.32)	7 (0.31)
Personality disorders	305 (40.88)	39 (1.75)

**Table 4:** The distribution of co-morbid disorders in case and control families.

	N (%)			
	Case families	Control families	p	V
Substance use disorders				
Fathers	45 (6.11)	47 (2.15)	<0.001	0.10
Mothers	33 (4.44)	30 (1.35)	<0.001	0.09
Siblings	14 (1.60)	10 (0.34)	<0.001	0.07
Offspring	0 (0.00)	0 (0.00)	-	-
Total	92 (3.74)	87 (1.12)	<0.001	0.09
Schizophrenia				
Fathers	8 (1.09)	14 (0.64)	n.s.	0.02
Mothers	8 (1.08)	8 (0.36)	<0.05	0.04
Siblings	12 (1.37)	8 (0.27)	<0.001	0.06
Offspring	0 (0.00)	0 (0.00)	-	-
Total	28 (1.14)	30 (0.39)	<0.001	0.04
Bipolar disorders				
Fathers	5 (0.68)	10 (0.46)	n.s.	0.01
Mothers	16 (2.15)	18 (0.81)	<0.05	0.05
Siblings	3 (0.34)	2 (0.07)	n.s.	0.03
Offspring	0 (0.00)	0 (0.00)	-	-
Total	24 (0.98)	30 (0.39)	<0.05	0.03
Depression				
Fathers	17 (2.31)	24 (1.10)	<0.05	0.04
Mothers	38 (5.11)	39 (1.75)	<0.001	0.09
Siblings	19 (2.18)	22 (0.75)	<0.05	0.06
Offspring	0 (0.00)	0 (0.00)	-	-
Total	74 (3.01)	85 (1.10)	<0.001	0.07
Anxiety disorders				
Fathers	9 (1.22)	13 (0.59)	n.s.	0.03
Mothers	36 (4.85)	17 (0.76)	<0.001	0.13
Siblings	13 (1.49)	8 (0.27)	<0.001	0.07
Offspring	0 (0.00)	0 (0.00)	-	-
Total	58 (2.36)	38 (0.49)	<0.001	0.08
Eating disorders				
Fathers	0 (0.00)	0 (0.00)	-	-
Mothers	3 (0.40)	4 (0.18)	n.s.	0.02
Siblings	5 (0.57)	10 (0.34)	n.s.	0.02
Offspring	0 (0.00)	0 (0.00)	-	-
Total	8 (0.33)	14 (0.18)	n.s.	0.01
Personality disorders				
Fathers	39 (5.30)	35 (1.60)	<0.001	0.10
Mothers	62 (8.34)	53 (2.38)	<0.001	0.13
Siblings	25 (2.86)	19 (0.65)	<0.001	0.09
Offspring	0 (0.00)	0 (0.00)	-	-
Total	126 (5.12)	107 (1.38)	<0.001	0.11

**Fig 1:**
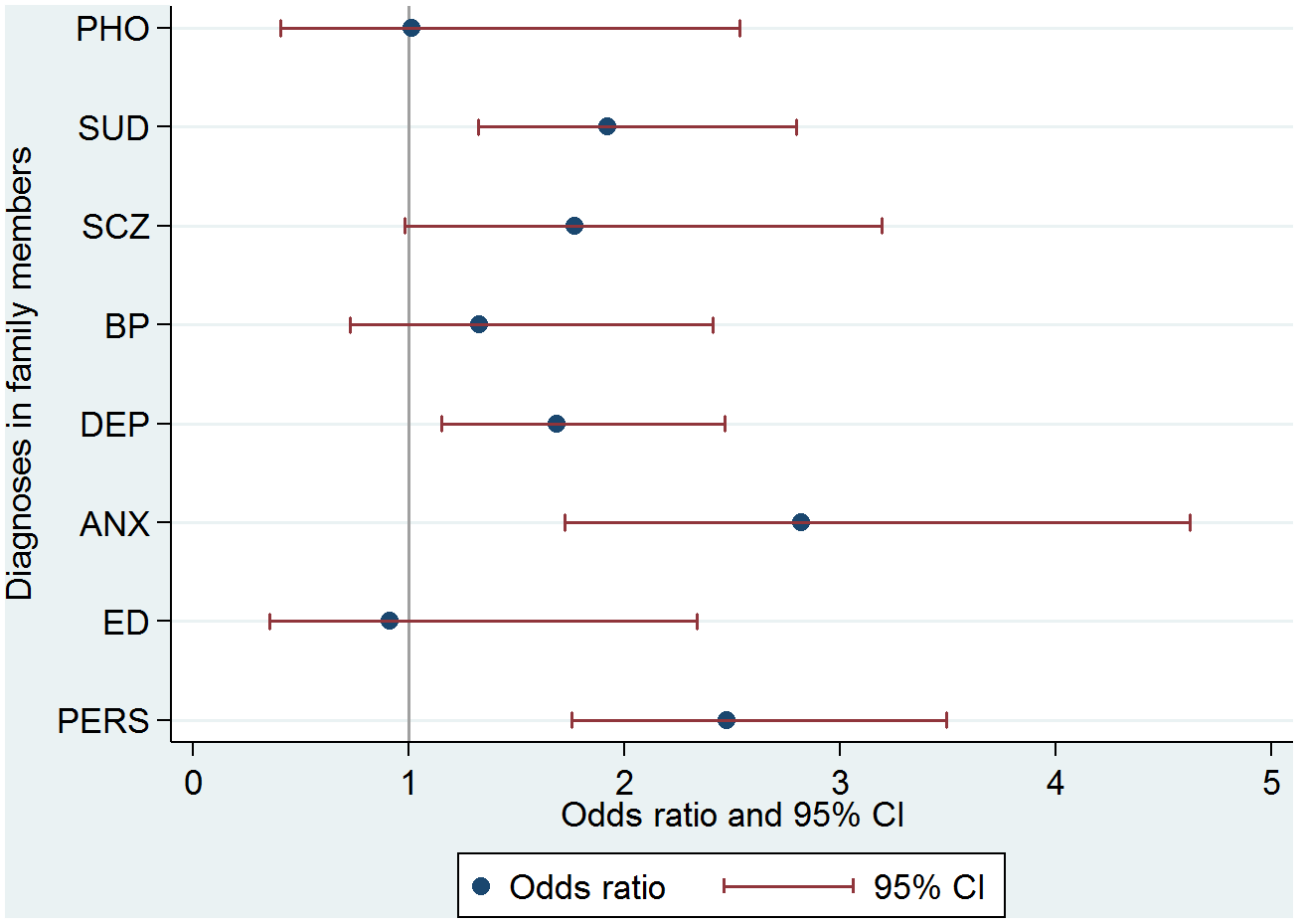
Associations of PHO in case-probands versus control-probands with mental disorders in first-degree family members. SUD = Family Substance use disorders, SCZ = Family Schizophrenia, BP = Family Bipolar disorders, DEP = Family Depression, ANX = Family Anxiety disorders, ED = Family Eating disorders, PERS = Family Personality disorders

Findings from the conditional logistic regression analysis determining the association of PHO in case-probands vs. controls-probands with either PHO or other mental disorders in the total number of family relatives is illustrated in Fig 1. Compared to the control-probands, PHO in the case-probands were significantly associated with family substance use disorders (SUD) (OR = 1.92, CI = 1.32–2.79), family depression (DEP) (OR = 1.69, CI = 1.15–2.47), family anxiety disorders (ANX) (OR = 2.82, CI = 1.72–4.62) and family personality disorders (PERS) (OR = 2.47, CI = 1.75–3.49). The results suggest that family schizophrenia (SCZ) (OR = 1.77, CI = 0.98–3.19), family bipolar disorders (BP) (OR = 1.33, CI = 0.73–2.41), family eating disorders (ED) (OR = 0.91, CI = 0.35–2.34) and family phobia (PHO) (OR = 1.01, CI = 0.40–2.53) may contribute to the risk of PHO in the case-probands, but the confidence intervals were too wide to rule out the possibility of no effect.

The results of multinomial logistic regression considering PHO as a comorbid condition (N = 619) in the case-probands vs. a pure condition without any further comorbidity (N = 127) and the controls without PHO are displayed in Table 5. In this analysis, parental age at birth of the case-proband and the mental disorders in the family members served as risk factors. The mental disorders in family members are measured by a binary variable that is one if at least one family member has a diagnosis that is not PHO and zero otherwise. Due to the rather small numbers of the various mental disorders in the family members of case-probands with pure PHO and also in the matched control families, these numbers had to be collapsed into a single variable of any comorbid disorder in family members. The model was adjusted for the matched variables age, sex and region of residence. The results show, that if a family member has a comorbid disorder, then the relative risk for a proband getting comorbid PHO is expected to increase by a factor of 3.80, given the other variables in the model are held constant. A Wald test was performed to test if the effect of any disorders except PHO among family members in predicting comorbid PHO among case-probands vs. case-controls equals the effect of any disorders except PHO among family members in predicting pure PHO among case-probands vs. control-probands. The test showed that at a significance level of 0.05 the effects are statistically different from each other (chi2(1) = 4.94, p = 0.0263). That is, if a family member has any comorbid diagnosis, then the relative risk of a case-proband getting comorbid PHO is significantly higher than the relative risk of a case-proband getting pure PHO compared to the control-probands. However, the confidence intervals overlap, so the possibility of equal relative risk ratios for pure and co-morbid PHO cannot be ruled out.

**Table 5:** Multinomial logistic regression with three groups of probands as the outcome variable (case-probands having either comorbid or pure phobia and phobia-free controls as the reference group).

	RRR	SE	p	95% CI
*Controls*	(reference)			
*Comorbid phobia*				
Any other disorder than PHO among family members	3.80	0.43	<0.001	3.05–4.73
Maternal age at birth 35 or over	1.08	0.21	n.s.	0.74–1.57
Paternal age at birth 35 or over	0.88	0.12	n.s.	0.68–1.15
*Pure phobia*				
Any other disorder than PHO among family members	2.24	0.52	<0.001	1.43–3.52
Maternal age at birth 35 or over	1.17	0.47	n.s.	0.54–2.55
Paternal age at birth 35 or over	0.60	0.18	n.s.	0.33–1.07

Table 6 shows the results of a mixed logistic regression which has the purpose of estimating the family load. The matched variables are included in order to take the matching into account. Whereas sex and year of birth were significant additional risk factors, month of birth and urbanization were not. The random effect contributed with 0.0013 percent of the total variance and a likelihood ratio test comparing this model to a model without a random effect was not significant (Likelihood-ratio test of contribution to variance = 0: χ^2^ = 0.001, p = 0.491), meaning that the family load component does not need to be included in the analyses.

**Table 6:** The effect of additional explanatory variables on the manifestation of PHO.

	Odds ratio	SE	p	95% CI
Sex				
Female	1.00			Reference
Male	0.59	0.05	<0.001	0.50–0.69
Month of birth				
January	1.00			Reference
February	1.14	0.22	n.s.	0.78–1.66
March	0.97	0.19	n.s.	0.66–1.42
April	0.91	0.18	n.s.	0.62–1.33
May	1.03	0.19	n.s.	0.72–1.50
June	1.15	0.22	n.s.	0.79–1.66
July	1.06	0.20	n.s.	0.73–1.52
August	1.01	0.19	n.s.	0.70–1.46
September	1.23	0.22	n.s.	0.86–1.75
October	1.16	0.22	n.s.	0.79–1.68
November	1.00	0.20	n.s.	0.68–1.49
December	1.03	0.20	n.s.	0.71–1.51
Year of birth	1.06	0.00	<0.001	1.05–1.06
Degree of urbanisation				
City of Copenhagen	1.00			Reference
Other regions	0.95	0.09	n.s.	0.80–1.14
Standard deviation	0.01	0.03		0.00–211.31
Contribution to variance	1.32*10^−5^	0.00		0.00–1.00

**Fig 2:**
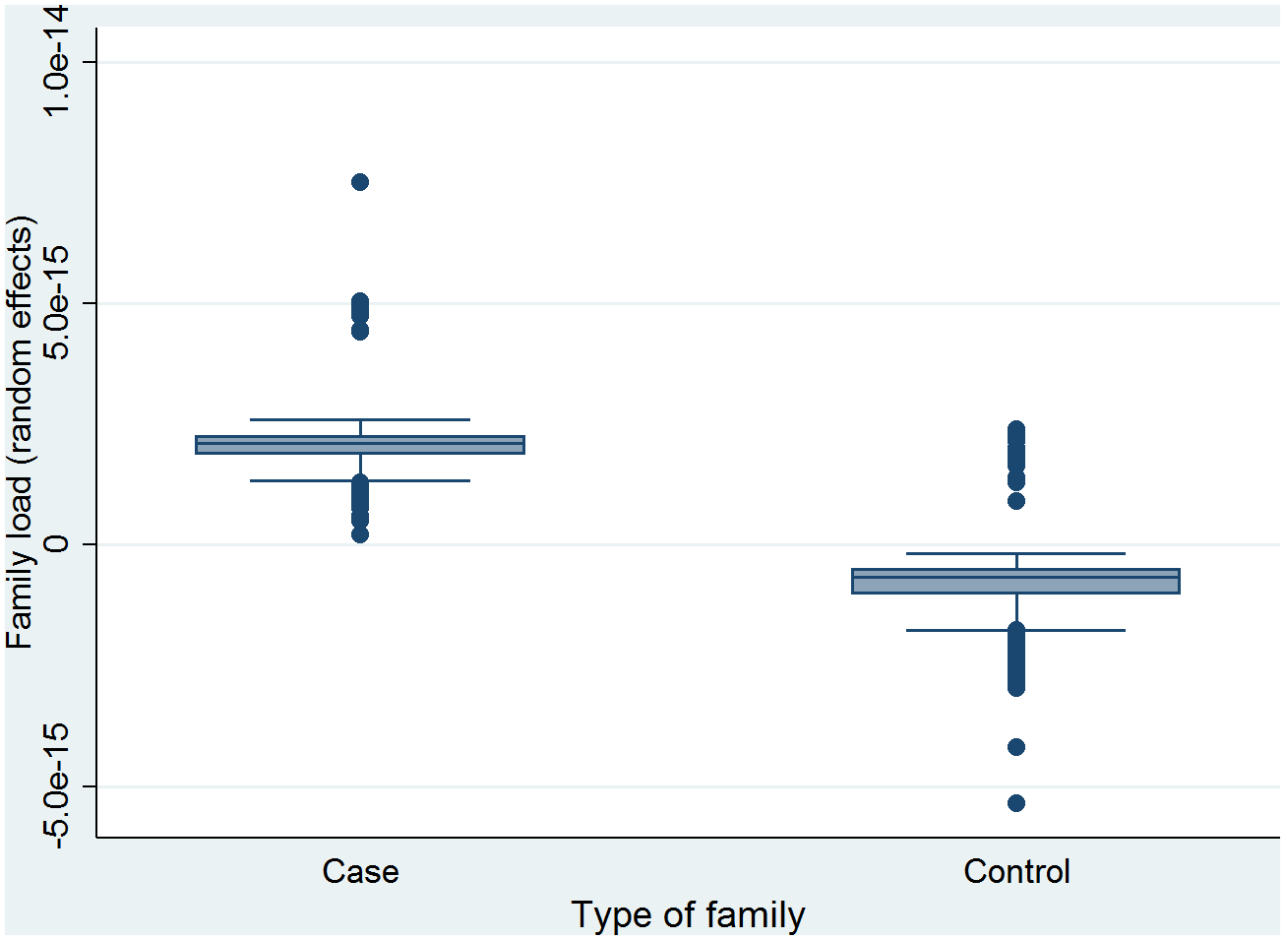
Family load components of PHO in case-probands and controls.

The box plot in Fig 2 displays the family load components estimated on the basis of mixed logistic regression. Year of birth, month of birth, sex and degree of urbanization make up the fixed effects of the mixed logistic regression model, while the random effect reflects the correlation between family members, i.e. the risk level of PHO for each family. The family load is defined as the random effect which is shown in Fig 2. It can be seen that case families had a significantly higher family load component than control families, meaning that family aggregation explains a larger part of the variance in case families than in control families. The two-sample Wilcoxon rank-sum test for testing the family load among males and females separate for the case and the control-probands showed that there was a significant difference in the family load between females and males among both cases and control-probands indicating a higher load in males (Case-probands: Females N = 500, rank sum = 169837, expected = 186750 vs. males N = 246, rank sum = 108794, expected = 91881; z = -6.11, p<0.001. Control-probands: Females N = 1494, rank sum = 1505070, expected = 1665810 vs. males N = 735, rank sum = 980266, expected = 819525; z = -11.25, p<0).

The question of whether or not PHO emerges earlier in case family members than control family members was investigated using Cox regression with shared frailty. As described above, this method estimates the family load component as a random effect in a similar way to mixed logistic regression. However in this analysis the family load component (frailty) is estimated in relation to the time of PHO onset. All individuals were followed from birth or April 1^st^ 1969 until date of diagnosis of PHO, date of death, or December 10^th^ 2009 where data was censored if PHO or death had not occurred. The predictors included in the model were the matched variables, i.e. sex, degree of urbanization, year of birth, and month of birth. The results indicated that none of the predictors, except for year of birth and males were significant. The hazard ratio (HR) showed, that later year of birth was a risk factor (HR = 1.14, p<0.001) and that being male (HR = 0.29, p<0.05) lowered the risk. The variance of the frailty was 16.56 which was not significant (p = 1.00) indicating that the case relatives did not develop PHO earlier than the control relatives.

## Discussion

The primary aim of this study was to evaluate the familial aggregation of specific phobia among first-degree relatives of affected probands. We found that PHO occurred more often in case than in control families, in particular, in mothers and siblings. The higher rates of these disorders among first-degree relatives of affected probands support results reported by previous studies [16–22] which found family aggregation of specific phobic disorders. This familial aggregation may be explained by several factors including behaviour modelling, shared environment, and genetic factors. The present study design does not allow us to disentangle these various factors.

After demonstrating in bivariate analyses the rather high rate of almost all mental disorders in the various family members with an exception of fathers and offspring, the conditional logistic regression analysis showed that substance use disorders (SUD), anxiety disorders (ANX), depression (DEP) and personality disorders (PERS) in the family were significantly associated with phobic disorder in the case-probands. This cross-aggregation between phobic disorders and depressive disorders, other anxiety disorders and personality disorders is a novel finding of the present study and may be due to shared familial risk factors underlying these mental disorders. The associations with phobia (PHO), schizophrenia (SCZ), bipolar disorders (BP) and eating disorders (ED) had too wider confidence intervals to rule out the possibility of no effect, but all the CI’s included mostly values above one, i.e. they could be risk factors as well.

Considering the fact that PHO occurred most commonly with other comorbid disorders in the case-probands, the family aggregation had to be studied separately in case-probands with either comorbid or pure manifestations of PHO. The findings after controlling for various mental disorders comorbid to PHO indicated that some of the family transmission was not due to the PHO itself but rather due to various other mental disorders in family members having a significant impact on the morbidity of the case-probands. These findings point to rather complex patterns of transmission of disorders which, unfortunately could not be studied more in detail in the present study due to the rather small numbers of comorbid disorders in the families of probands with pure PHO and their matched controls did not allow for separate analyses of the various disorders but rather, forced us to combine these disorders in an aggregated category of any comorbid disorder in family members.

The finding that females reported higher rates of specific phobia than males is well known and supports the findings of large scale epidemiological studies [4,5]. In addition, recent year of birth was a further risk factor indicating a period effect, which may be due to a more thorough registration of patient data in the recent years or other unknown factors that are not yet fully understood in terms of an increased risk of the very young patients in this large cohort. Finally, it was found that region of residence was not related to the first diagnosis of PHO. Obviously, urbanization with potentially underlying stress factors or stronger help-seeking attitudes does not play a significant role in the manifestation of PHO, which is in contrast to our findings with the same approach in SCZ [27], ANX [29] but in line with findings in BP [28], OCD [30], and AN [32].

After considering the various additional explanatory variables, the analyses indicated that the family load estimate explained only 0.0013% of the variance in the manifestation of disorder in the case-probands reflecting both the non-specificity of psychiatric inheritance and the relevance of other factors. Although the higher rates of these disorders among first relatives of affected probands support results reported by previous studies [16–22], the small amount of only 0.0013% explained variance of the family load of PHO in the present study based on the largest and most representative sample so far indicates that the family aggregation of PHO is smaller than assumed in previous studies. In addition, our finding has to be considered in perspective with our other family aggregation studies revealing a 23% rate of explained variance in schizophrenia [27], a 20% rate in bipolar disorders [28], a 12% rate in anxiety disorders [29], a 6% rate in obsessive compulsive disorders [30], and almost 0% rate in anorexia nervosa [32]. Using the twin study approach, the Virginia Twin Study found modest to moderate heritability estimates between 0.10 and 0.36 for phobic disorders [38]. Likewise, another twin study [39] suggested that phobic disorders are mainly caused by environmental factors. In conclusion, results of our approach and the twin study approach suggest a rather modest importance of familial factors in the etiology of phobic disorders.

Advantages of the study include surveillance of a large population sample, an extended period of observation, a data set covering three generations, and matching of cases and controls on potentially confounding variables. There are several limitations. First, a certain number of cases of illness treated by private services have not entered the analysis, although private care is uncommon in Denmark. Secondly, the rather late registration of outpatients in the DCPRR since 1995 only may have resulted also in an under-representation of the true number of patients with PHO. Thirdly, given that mental disorders in the data set are determined by treatment seeking, it can be assumed that some cases of both proband and familial illness remain “undetected,” and this may pertain to PHO as well. On the other hand, having a mentally ill relative might have increased the chance of seeking professional assistance and, thus, the chance of registration. Finally, there is no independent verification of the accuracy of diagnoses entered in the DCPRR, although prior quality checks on the DCPRR suggest that diagnostic validity is high across a range of disorders [40–43].
